# Intimate Partner Violence May Be One Mechanism by Which Male Partner Socioeconomic Status and Substance Use Affect Female Partner Health

**DOI:** 10.3389/fpsyt.2018.00160

**Published:** 2018-05-08

**Authors:** Shervin Assari, Rohan D. Jeremiah

**Affiliations:** ^1^Department of Psychiatry, University of Michigan, Ann Arbor, MI, United States; ^2^Center for Research on Ethnicity, Culture and Health, School of Public Health, University of Michigan, Ann Arbor, MI, United States; ^3^Division of Community Health Sciences, School of Public Health, University of Illinois at Chicago, Chicago, IL, United States; ^4^HIV/AIDS, Substance Abuse and Trauma Training Program, University of California, Los Angeles, Los Angeles, CA, United States

**Keywords:** intimate partner violence, gender, mental health, self-rated health, socioeconomic status, substance use

## Abstract

**Background:** Although male partners' socioeconomic status (SES) and substance use is associated with worse health of female partners, the mechanism behind this link is still unknown.

**Objectives:** To investigate whether intimate partner violence (IPV) is a mechanism by which male partners' SES and substance use influence female partners' self-rated health (SRH) as victims and survivors of IPV.

**Materials and Methods:** Fragile Families and Child Wellbeing Study (FFCWS) is an ongoing population-based cohort. Male and female partners' SES, anxiety, depression, and substance use, and their relationship status were measured at baseline. IPV victimization was also asked among female partners' at baseline. Female partners' subjective health was measured 3 times (baseline−1998, 3 years later−2001, and 5 years later−2003). Using AMOS, we fitted two structural equation models (SEM) for data analysis. In *Model 1* we tested direct paths from male partners' SES and mental health to female partners' SRH, in the absence of IPV. In the *Model 2* we conceptualized female partners' IPV victimization between male partners' SES and mental health and female partners' SRH. In both models we controlled for the effect of female partners' SES and mental health.

**Results:** In *Model 1*, male partners' poor SES and substance use were associated with worse trajectory of SRH of female partner. In *Model 2*, male to female IPV was the mechanism by which male partners' SES and substance use were associated with female partners' SRH.

**Conclusions:** IPV is one of the mechanisms by which male partners' SES and substance use can influence female partners' health. That is, IPV may operate as a vehicle by which male partners' social and psychological risk factors impact female partners' health. Thus, this study demonstrates how male partners' socio-ecological risk factors such as low SES and substance use impact female partners' health. Therefore, there is a need for broader socio-ecological approach to IPV prevention and intervention that recognizes the relationship between male partners' risk factors and their female partners' health outcomes. Such approach can inform prevention and treatment of IPV and enhance partner wellbeing.

## Background

Intimate Partner Violence (IPV) is a serious public health problem (1). Although both males and females may use violence against their partners, male to female IPV is more common than female to male IPV (2). Based on the World Report on Violence and Health, up to 69% of women experience physical abuse during their lifetime (3). In the United States, 29% of women experience physical violence, rape, and/or stalking by an intimate partner, 24% experience severe physical violence by an intimate partner, and 15% become injured as a result of IPV (4–6).

Exposure to IPV is associated with poor psychological and physical health (7). IPV victims also report worse overall health and sense of well-being, which hinders their ability to live a life without pain and suffering. In a study, quality of life was worse across physical health, social relationship, environment, and psychological health domains in women who had experienced IPV (8). Other studies have also documented poor quality of life of victims of physical, psychological and sexual IPV (9–11).

Most of the research on male to female IPV has focused on either the perpetrator or the victim (12–14), with minimum research on if male to female IPV is the actual mechanism by which male SES influence females' sense of well-being. The role of male to female IPV as a mechanism by which male partner socio-economic status (SES) factors and mental health influences female partner health is not fully investigated (15). The well-established associations identified during the last 30 years include a link between SES and IPV (16). Research has shown that separated or divorced women, those with unemployed partners, and people with low household income experience more abuse (17). Analysis of the National Crime Victimization Survey data showed that young women (age 19–29) in low-income families (under $10,000) are more likely than other women to experience IPV (18).

Another segment of IPV research that has not been fully investigated is whether IPV is a mechanism by which male partner mental health impacts female partner well-being. A few studies have shown that early-onset of mental disorders may increase risk of IPV perpetration among men (19–21). In one study, premarital Major Depressive Disorder (MDD), Generalized Anxiety Disorder (GAD), antisocial behavior, and non-affective psychosis predicted IPV perpetration among married or cohabiting men in the United States general population (19). Substance use increases risk of IPV perpetration (22), while alcohol misuse is one of the most established risk factors for IPV perpetration (22–25). Several female IPV victims report that their male partners had consumed alcohol in the episode of violence (26). Involvement in illicit drug use also increases the risks of IPV perpetration (27). As a result, World Health Organization (WHO) has used the term drug-related IPV (27). Again, it is still unknown whether IPV can operate as one of the potential mechanisms by which male partners' mental health problems impact female partners' health and sense of well-being.

The current study tested the role of IPV as a mechanism by which male partners' poor SES and substance use affect poor well-being of female partner. We conceptualized male to female's IPV as a bridge connecting male partners' SES and substance use to well-being of female partner.

## Methods

### Design and setting

Fragile Families and Child Wellbeing Study (FFCWS) is an ongoing population-based cohort of 4,898 male and female partners, started in 1998. Participants were male and female partners who had a newborn in 20 U.S. cities with populations of 200,000 or more. Baseline data were collected in 75 hospitals across 20 cities. From all participants, 3,712 couples were unmarried and 1,186 couples were married at baseline. A detailed description of the FFCWS sampling strategy and interview protocol is available (28).

### Ethics

The study protocol was approved by Institutional Review Board Committees at Princeton University and Columbia University. Verbal and written informed consent was obtained from participants at each interview, and all participants were compensated for their involvement in the study.

### Process

Data were collected during core interviews and the add-on in-home interviews (more in-depth interviews that collected additional data). Male and female partners were interviewed at baseline-1998 (near the time of the target child's birth), 1 year later (Wave 2), 3 years later (Wave 3) and 5 years later.

The Fragile Families and Child Wellbeing Study has oversampled non-married couples (28). As non-marital unions are at greater risk for relationship instability, a large number of male partners were not living with female partners in Waves 2 or 3. For instance, by the time the study target child was 3 years old, fewer than half of male partners were residing in the home.

### Measures

Most variables were based on paternal self-report. Male to female IPV was based on female partners' self-report.

### Outcomes

#### Male to female physical IPV

Physical IPV was assessed by asking female partners four questions, on a 3-point scale (“*never*,” “*sometimes*,” or “*often*”), regarding how often the male partner carried out behaviors toward the female partner, (e.g., slapping, kicking, hitting with fist, hitting with an object). Items were adapted from the Conflict Tactics Scale (CTS-2) for adults (29, 30). The original and revised Conflict Tactics Scales (29, 30) have been the most common research measures of domestic violence, and the 1996 version includes separate measures of psychological dimensions, physical violence, sexual violence and financial control. The physical violence items of the CTS are still the most widely used approach to assessing levels of domestic violence (31).

### Predictors

#### Major depressive disorder (MDD) and generalized anxiety disorder (GAD)

The Composite International Diagnostic Interview - Short Form (CIDI - SF), Section A (32) was used to measure MDD and GAD. The CIDI-SF is a standardized instrument that is consistent with the criteria set forth in the Diagnostic and Statistical Manual of Mental Disorders – Third Edition – Revised (DSM-III-R) (33). This instrument has good reliability and validity for measurement of MDD and GAD (32). The CIDI-SF uses the criteria set forth in the Diagnostic and Statistical Manual of Mental Disorders (DSM-IV) to determine the probability that the respondent would be diagnosed with MDD, GAD, and other psychiatric disorders if given the full CIDI interview. MDD is indicated by feelings of depression or anhedonia experienced for most of the day, every day, for at least 2 weeks. Participants were classified as likely to have MDD if they endorsed the screening items and 3 or more depressive symptoms (e.g., losing interest, feeling tired, change in weight) (0 = no, 1 = yes). GAD is indicated by a period of 6 months or more when an individual feels excessively worried or anxious about more than one thing, more days than not, and has difficulty controlling their worries. Common symptoms of GAD include being keyed up or on edge, irritability, restlessness, having trouble falling asleep, tiring easily, difficulty concentrating and tense or aching muscles. Subjects were classified as having GAD if they met full diagnostic criteria based on the CIDI-SF (0 = no, 1 = yes).

#### Substance use

Tobacco use, alcohol use and illicit drug use were measured. This measure was operationalized as a latent factor in this study. All of the drug and alcohol abuse data used in this analysis are drawn from female partner's reports. This is because of considerable proportion of missing data in male partners' report data (primarily due to attrition and/or refusal to participate among male partners who do not live with their child). Thus, we relied only on female partners' reports.

#### Self-rated health (SRH)

Female respondents were asked to classify their self-rated health as excellent, very good, good, fair, or poor. We treated SRH as a continuous score. SRH is coded from 1 (excellent) to 5 (poor), with higher values indicative of worse SRH. Poor SRH is a strong predictor of mortality (34–36).

#### Socioeconomic status (SES)

All following SES variables were measured at the baseline interview (Wave 1): age, education level, income, minority status, and relationship status. Partners' demographic factors included household income, age at the time of the child's birth, education level (1 = less than high school, 2 = high school degree or GED, 3 = some college/technical school or higher), and relationship status (1 = married, 2 = cohabiting, 3 = not married or cohabiting).

### Analysis plan

We used SPSS 20 for our univariate and bivariate analysis. The Pearson correlation test was used for bivariate associations between male partners' SES, and male partners' mental health, female partners' SES, IPV, and female partners' SRH. We used AMOS for multivariable analysis. We fitted two structural equation models (SEM) for data analysis.

*Model 1* included direct paths from male partners' SES (a latent factor) and male partners' substance use to female partners' baseline and trajectory of SRH, while the effect of female partners' SES and mental health is controlled.

In *Model 2*, we hypothesized that male partners' SES and male partners' substance use would be associated with IPV victimization among female partners, and that female partners' IPV victimization would be in turn associated with female partners' trajectory of SRH. In both models, female partners' SES and mental health (i.e., depression, anxiety, and substance use) were control variables.

The chi-square test, comparative fit index (CFI), root mean square error of approximation (RMSEA), and chi-square to degree of freedom ratio were considered as fit indices. A CFI of higher than 0.90, RMSEA of lower than 0.08 and chi-square test to degree of freedom ratio less than 4 were indicative of good fit (37). Although variables measured at baseline data did not have considerable missing values, variables measured at other waves had more missing values. We did not impute the data.

### Missing data

AMOS uses the Full Information Maximum Likelihood (FIML) method to handle missing data. So, all available data from all participants contribute to SEM models, even in the presence of missing data due to loss to follow up. This approach reduces selection bias due to complete data analysis. This approach is in contrast to Listwise or case deletion (deleting any case that has any missing value), as it preserves all cases that enter to the original study (38).

## Results

### Bivariate analysis

Based on bivariate analysis, female SES, GAD, MDD, and substance use were significantly associated with female SRH. Education, relationship, marriage status, and cohabitation status all had marginally significant association with baseline SRH. Female partners' poverty index, Black race, and GAD were significantly associated with change of SRH over time. Age, employment, MDD and substance use all had marginally significant association with SRH over time.

### Unconditional model of the outcome

Unconditional model of baseline and slope of female partners SRH showed an excellent fit to the data. There was a strong and negative correlation between baseline and trajectory of SRH among female partners (*B* = 0.67, *P* < 0.001), suggesting that female partners who started with a higher SRH at baseline were those who experienced a better trajectory of SRH over time. Baseline (variance = 12.36, *SE* = 0.97, *P* < 0.001) and slope (variance = 1.84, *SE* = 0.20, *P* < 0.001) of SRH of the female partners showed significant variance, suggesting that their variance can be used as an outcome in the models. Thus, we considered baseline and slope as outcomes in the following two models (*Model 1* and *Model 2*).

### Model 1

In *Model I*, we tested direct paths from male partners' SES, MDD, GAD, and substance use to baseline and slope of female partners' SRH. The model showed a good fit [Chi-square = 14437.514, Probability level < 0.001, Degrees of freedom = 348, CFI = 0.936, RMSEA = 0.090]. Our findings suggested that while the effect of female partners' SES and mental health are controlled, male partners' MDD and GAD do not have any association with baseline or slope of SRH among female partners. However, male partner SES and SU were directly associated with both baseline and slope of SRH among female partners in this model (Table 1).

**Table 1:** Descriptive statistics in male and female partners in the study.

**Characteristics**		
	**Mean**	***SD***
**MALE PARTNER**		
Age	19.87	16.51
Income	29398.74	34282.49
Education	1.89	1.39
	*n*	%
**RACE**		
White, non-hispanic	894	18.3
Black, non-hispanic	2,407	49.1
Hispanic	1,354	27.6
Other	216	4.4
**FEMALE PARTNER**		
Age	25.25	6.10
Income	31987.51	31567.26
Education	2.10	1.03
	*n*	%
**RACE**		
White, non-hispanic	1,030	21.0
Black, non-hispanic	2,326	47.5
Hispanic	1,336	27.3
Other	194	4.0
**SRH**		
SRH 1	2.11	0.95
SRH 2	2.23	1.06
SRH 3	2.25	1.05
SRH 4	2.36	1.03

*SRH, Self-rated health*.

### Model 2

In *Model 2*, we tested the full model in which female partner IPV victimization was conceptualized as an intermediate mechanism that links male partners' predictors to female partners' SRH. The model showed a good fit (Probability level = 0.000, Degrees of freedom = 474, CFI = 0.954, RMSEA = 0.080). Based on this model, there were significant paths from male partners' MDD and GAD (in addition to SES and substance use) to female partners' IPV victimization. Female partner IPV victimization was also associated with SRH among female partners (Table 2).

**Table 2:** Summary of *Model 1* that tests the associations between male and female partners' SES, mental health and female partners' self-rated health in the absence of female IPV victimization in the model.

**Predictor**		**Outcome**	**Standardized beta**	***SE***	***P***
Female partner age	→	Poor health-Slope	0.015	0.004	0.674
Female partner black	→	Poor health-Slope	−0.016	0.082	0.757
Female partner hispanics	→	Poor health-Slope	−0.115	0.061	0.003
Female partner other races	→	Poor health-Slope	−0.075	0.115	0.017
Female partner relation status	→	Poor health-Slope	−0.090	0.054	0.010
Female partner cohabitation	→	Poor health-Slope	−0.157	0.043	<0.001
Female partner anxiety	→	Poor health-Slope	0.026	0.120	0.367
Female partner depression	→	Poor health-Slope	0.061	0.064	0.036
Female partner substance use	→	Poor health-Slope	0.111	0.073	0.004
Female partner age	→	Poor health-Baseline	−0.015	0.003	0.102
Female partner black	→	Poor health-Baseline	0.026	0.059	0.064
Female partner hispanics	→	Poor health-Baseline	0.054	0.044	<0.001
Female partner other races	→	Poor health-Baseline	0.014	0.083	0.089
Female partner relation status	→	Poor health-Baseline	0.004	0.039	0.644
Female partner cohabitation	→	Poor health-Baseline	0.037	0.031	<0.001
Female partner anxiety	→	Poor health-Baseline	0.034	0.087	<0.001
Female partner depression	→	Poor health-Baseline	0.037	0.046	<0.001
Female partner substance use	→	Poor health-Baseline	0.015	0.053	0.140
Male partner age	→	Poor health-Baseline	0.056	0.003	0.016
Male partner black	→	Poor health-Baseline	0.024	0.063	0.149
Male partner hispanics	→	Poor health-Baseline	0.044	0.050	<0.001
Male partner other races	→	Poor health-Baseline	0.013	0.084	0.133
Male partner anxiety	→	Poor health-Baseline	−0.062	0.057	0.607
Male partner depression	→	Poor health-Baseline	0.069	0.057	0.566
Male partner substance use	→	Poor health-Baseline	0.288	0.034	0.001
Male partner substance use	→	Poor health-Slope	0.326	0.046	0.326
Male partner depression	→	Poor health-Slope	0.683	0.079	0.139
Male partner other races	→	Poor health-Slope	−0.114	0.117	<0.001
Male partner hispanics	→	Poor health-Slope	−0.051	0.069	0.249
Male partner anxiety	→	Poor health-Slope	−0.199	0.079	0.666
Male partner age	→	Poor health-Slope	−0.083	0.004	0.351
Male partner high SES	→	Poor health-Baseline	−0.350	0.275	<0.001
Male partner high SES	→	Poor health-Slope	−0.166	0.371	0.616
Male partner black	→	Poor health-Slope	0.054	0.087	0.391

The results suggest that in the absence of considering IPV as an intermediate mechanism, male partners' MDD and GAD do not show an association with female partners' SRH. However, when we take into account IPV as a possible mechanism, male partners' MDD and GAD influence female partners' SRH through IPV victimization of the female partner. In contrast, to document the association between male partners' SES and substance use and female partners' SRH, there is no need to consider IPV as an intermediate factor.

## Discussion

The current study showed two findings. First, male partners' poor SES and alcohol abuse were associated with worse trajectory of SRH among female partner. Second, IPV may be a mechanism by which male partners' psychosocial factors such as SES and substance can influence female partners' mental health outcomes.

These findings emphasize the relevance of socioecological models for predicting and contextualizing the risks, occurrence and impact of IPV (39). Our finding reinforces the basis of the social ecological model (SEM) of health promotion that emphasizes a broader understanding of among the individual, relationship, community and societal factors that enable violence. Our study establishes a clear understanding that contextual factors male perpetrator such as his SES and substance use increase the health consequences among female IPV victims and survivors (40–43). From such vantage point, we are able to assess that IPV is more than just an act of violence but can be situated with multi-factoral reasons that influences a person's health and wellbeing. SEM approach to IPV demonstrares how IPV is manifested and sustained beyond physical abuse and toward the mental health outcomes observed in our study. Therefore, it is important to consider the prevention and intervention of intimate partner violence have to conceptualized from multi-level approach to reduce the public health consequences (44).

Our study also reinforces ample evidence that substance use and substance use disorders are proven associations among male perpetrated IPV (45–47). Bennett and Williams (48) assert that “intimate partner violence cannot be well explained as a simple sequela of substance use” (p. 560); however, they identify that there is an apparent relationship between the two, and a number of studies point to how substance use can shape violence. For instance, cocaine and marijuana use are associated with increased odds of aggression perpetration (45), as is alcohol use (49). Furthermore, Stuart et al. (52) found 31% of men arrested for domestic violence met criteria for a drug use disorder and 53% met criteria for an alcohol use disorder. Substance use and abuse with IPV enables a degree of trauma for both perpetrators and victims that have consequences on their health outcomes. The risk of domestic violence among men is also linked to mental health symptomatology. Research has shown that antisocial personality disorder (ASPD) and borderline personality disorder (BPD) traits are associated with the perpetration of IPV (47, 50) Additionally, Holtzworth-Munroe and Stuart's (51) typologies of IPV perpetrators were defined, in part, by borderline/dysphoric (BD) and generally violent/antisocial (GVA) features, both of which are associated with increased rates of perpetration (38). Studies have shown that these are often predictive of who recidivates after participating in batterer intervention programs (BIP) (52, 53). Our finding shows that IPV is one reason why poor SES and substance abuse transcend beyond the male perpetrator and become a health issue for IPV-exposed female.

Our finding shows male partners' SES and substance abuse are associated with the trajectory of the female partners' SRH. Such finding provides additional context to the existing literature about the interplay between SES, substance use, IPV, and health (16, 54, 55). Perpetrator's SES does impact female partner health status. Such finding suggests that the effects of low SES and poor mental health of one partner stress to traumatize and impact the health of another partner. There is a call for evaluation of IPV in families and couples where male partner has low SES and substance use problem. Male partner SES and substance use may have implications for well-being of the other partner.

This research also supports the literature that suggests low SES and substance abuse increase risk of IPV within families (56–58). In our analysis, IPV is a mechanism by which low SES and substance use in male partner impacts female partners' health (See Table 3). This study proposes that female partners well-being should be seen in a relational view, and promotion of female partner well-being may require IPV prevention, particularly in the presence of substance use and low SES.

**Table 3:** Summary of *Model 2* that tests the associations between male and female partners' SES, mental health, IPV, and female partners' self-rated health conceptualizing female IPV victimization as an intermediate variable.

**Predictor**		**Outcome**	**Standardized beta**	***SE***	***P***
Male partner substance use	→	IPV victimization	−0.491	0.158	0.003
Male partner depression	→	IPV victimization	−0.807	0.270	<0.001
Male partner anxiety	→	IPV victimization	1.000	0.272	<0.001
Male partner other races	→	IPV victimization	−0.069	0.334	<0.001
Female partner hispanics	→	IPV victimization	−0.074	0.193	<0.001
Male partner age	→	IPV victimization	0.215	0.011	<0.001
Male partner high SES	→	IPV victimization	0.402	0.902	0.016
Male partner black	→	IPV victimization	−0.196	0.172	<0.001
Female partner age	→	Poor health-Slope	−0.041	0.004	0.111
Female partner black	→	Poor health-Slope	0.019	0.071	0.630
Female partner hispanic	→	Poor health-Slope	−0.129	0.052	<0.001
Female partner other races	→	Poor health-Slope	−0.065	0.100	0.005
Relation status	→	Poor health-Slope	−0.291	0.047	<0.001
Cohabitation	→	Poor health-Slope	−0.196	0.037	<0.001
Female partner anxiety	→	Poor health-Slope	0.050	0.104	0.021
Female partner depression	→	Poor health-Slope	0.063	0.055	0.004
Female partner substance use	→	Poor health-Slope	0.077	0.063	0.007
Female partner age	→	Poor health-Baseline	−0.012	0.003	0.166
Female partner black	→	Poor health-Baseline	0.023	0.059	0.082
Female partner hispanics	→	Poor health-Baseline	0.053	0.044	<0.001
Female partner other races	→	Poor health-Baseline	0.013	0.083	0.088
Relation status	→	Poor health-Baseline	0.012	0.039	0.165
Cohabitation	→	Poor health-Baseline	0.037	0.031	<0.001
Female partner anxiety	→	Poor health-Baseline	0.032	0.086	<0.001
Female partner depression	→	Poor health-Baseline	0.036	0.046	<0.001
Female partner substance use	→	Poor health-Baseline	0.015	0.053	0.121
Male partner age	→	Poor health-Baseline	0.055	0.003	0.015
Male partner black	→	Poor health-Baseline	0.021	0.063	0.189
Male partner hispanics	→	Poor health-Baseline	0.041	0.050	<0.001
Male partner other races	→	Poor health-Baseline	0.011	0.084	0.201
Male partner anxiety	→	Poor health-Baseline	−0.032	0.057	0.785
Male partner depression	→	Poor health-Baseline	0.047	0.057	0.683
Male partner substance use	→	Poor health-Baseline	0.283	0.034	<0.001
Male partner substance use	→	Poor health-Slope	0.783	0.041	0.002
Male partner depression	→	Poor health-Slope	1.000	0.068	0.001
Male partner other races	→	Poor health-Slope	−0.045	0.101	0.066
Male partner hispanic	→	Poor health-Slope	−0.002	0.060	0.957
Male partner anxiety	→	Poor health-Slope	−0.939	0.069	0.006
Male partner age	→	Poor health-Slope	−0.071	0.003	0.280
Female partner high SES	→	Poor health-Baseline	−0.341	0.195	<0.001
Female partner high SES	→	Poor health-Slope	−0.710	0.232	0.004
Male partner Black	→	Poor health-Slope	0.104	0.076	0.026
IPV victimization	→	Poor health-Slope	0.884	0.004	<0.001
IPV victimization	→	Poor health-Baseline	−0.033	0.003	<0.001

More inquiries are needed to better understand the complex links between SES, substance use, IPV, and well-being of the partners. More efforts are needed to understand how coping, communication, and conflict explain the effect of IPV on male partner characteristics on female partner well-being. Well-being of a partner should be seen as interdependent with the SES and behavioral profile of her partner, and the presence of violence in the relationship.

This work draws attention to male partners' psychosocial factors (e.g., SES and substance use) (57, 59, 60) as risk factors of IPV that in turn impacts the health and wellbeing of women. There is a need to consider SES and behaviors of men as perpetrators to improve the health outcomes and coping strategies of women. We have learned that though these elements may or may not impact the lives of women in the absence of IPV, when IPV is present, the linkage to a woman's health and wellbeing is impactful (41).

Our finding suggests that when considering ways to promote well-being of women, male partners' SES and substance use and associated male to female IPV should be considered. It is imperative that IPV prevention is delivered to low SES families with the history of substance use problems. In addition, programs should integrate ways to screen and treat anxiety, depression, and substance abuse of the male perpetrators.

A more careful consideration must be given into how IPV prevention should be regarded as a core element of programs that wish to enhance well-being of low SES families that have low SES and substance use problem. IPV victims should receive health evaluation.

## Limitations

**Figure 1:**
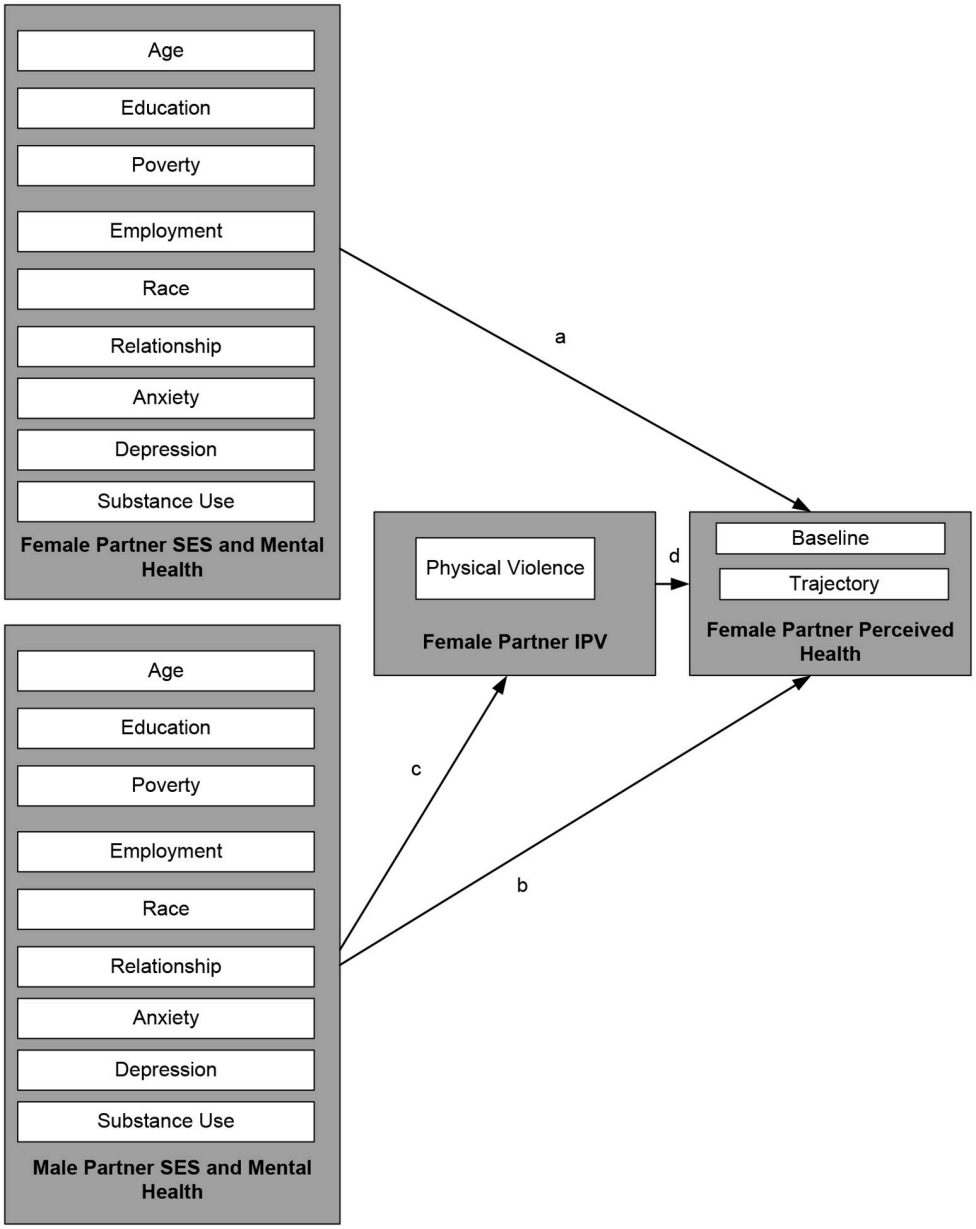
Conceptual model and modeling approach: In Model I, paths c and d were absent. In Model II, paths a, b, c, and d were tested.

The current study had several limitations. First, it only used a few items from CTS to measure IPV. Second, it only measured physical not psychological or sexual IPV. There are a number of unmeasured confounders such as physical health problems in female partner. In addition, the study was exclusively limited to heterosexual couples. Finally, we used female partners' reports of independent variables (See Figure 1). This decision was based on several reasons. First, we were only interested in male to female IPV, In addition, in the FFCWS, male and female partners' reports of the same variables do not have high concordance. As the FFCWS has enrolled an economically disadvantaged population which is composed of mostly unmarried couples, many households are headed by the female partner (single headed households), thus the female partners' reports may be considered more reliable and accurate than male partners' reports of the same constructs (14).

## Implication for health policy makers/practice/research/medical education

The study suggests that intimate partner violence may be a bridge between male partners' mental health to female partners' perceived health. The study highlights a need for multi-dimensional approach to the prevention of intimate partner violence as a strategy for health promotion of women who's male partner/spouse has poor mental health.

## Conclusion

While more than 30 years of IPV research has shown that SES and mental health problems increase risk of IPV, and IPV reduces sense of well-being, it was not known whether male to female IPV is the mechanism by which male SES and mental health problems influence female partner health and wellbeing. This study suggests that IPV, a public health challenge, is one reason why male partners' characteristics influence female partners' well-being. More research is needed to understand how these findings can inform prevention and treatment of IPV and to enhance wellbeing of partners. Screening and treatment of substance use among male partners may be an important component of prevention and treatment of IPV and enhancing female partners' partner wellbeing.

## Authors contributions

SA: design, analysis, early draft and also revision of the manuscript; RJ: literature review and discussion, early draft and revision of the manuscript draft.

### Conflict of interest statement

The authors declare that the research was conducted in the absence of any commercial or financial relationships that could be construed as a potential conflict of interest.
